# 3-Hydr­oxy-*N*′-(2-methoxy­benzyl­idene)-2-naphthohydrazide

**DOI:** 10.1107/S1600536809043086

**Published:** 2009-11-04

**Authors:** Yu-Mei Hao

**Affiliations:** a Department of Chemistry, Baicheng Normal University, Baicheng 137000, People’s Republic of China

## Abstract

In the title Schiff base compound, C_19_H_16_N_2_O_3_, the dihedral angle between the mean planes of the benzene ring and the naphthyl ring system is 0.8 (2)°. The mean plane of the hydrazide group forms dihedral angles of 2.0 (2) and 2.2 (2)°, respectively, with the mean planes of the benzene ring and the naphthyl ring system. A strong intra­molecular N—H⋯O hydrogen bond is present. In the crystal, inter­molecular O—H⋯O hydrogen bonds form chains along the *c* axis and help to provide stability in the crystal packing.

## Related literature

For the pharmaceutical and medicinal activities of Schiff bases, see: Dao *et al.* (2000 ▶); Sriram *et al.* (2006 ▶); Karthikeyan *et al.* (2006 ▶). For the coordination chemistry of Schiff bases, see: Ali *et al.* (2008 ▶); Kargar *et al.* (2009 ▶); Yeap *et al.* (2009 ▶). For the crystal structures of Schiff base compounds, see: Fun *et al.* (2009 ▶); Nadeem *et al.* (2009 ▶); Eltayeb *et al.* (2008 ▶); Hao (2009*a* ▶,*b* ▶). For bond-length data, see: Allen *et al.* (1987 ▶).
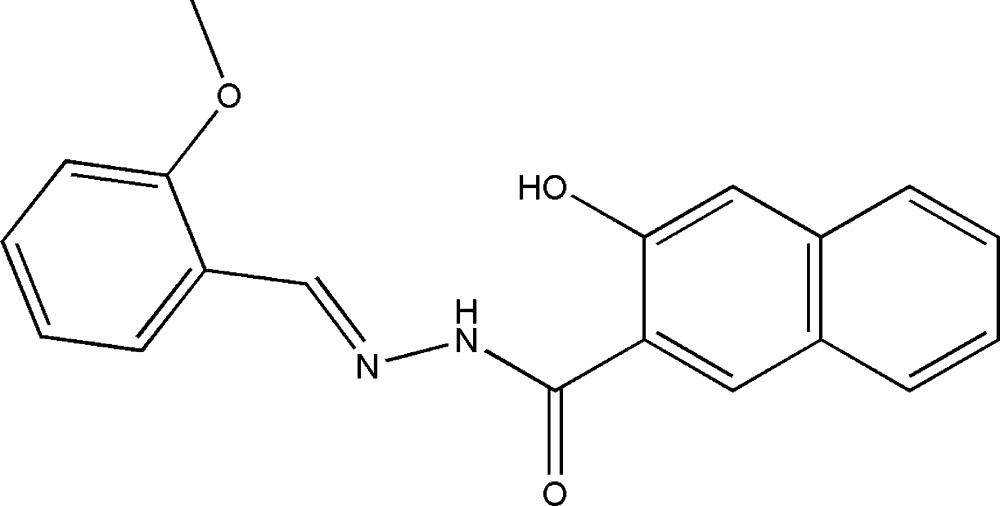


## Experimental

### 

#### Crystal data

C_19_H_16_N_2_O_3_
*M* *_r_* = 320.34
Monoclinic, 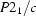

*a* = 7.4990 (6) Å
*b* = 15.4256 (13) Å
*c* = 13.3903 (12) Å
β = 96.709 (4)°
*V* = 1538.3 (2) Å^3^
*Z* = 4
Mo *K*α radiation
μ = 0.10 mm^−1^
*T* = 298 K
0.18 × 0.17 × 0.17 mm

#### Data collection

Bruker SMART CCD area-detector diffractometer
Absorption correction: multi-scan (*SADABS*; Sheldrick, 1996 ▶) *T*_min_ = 0.983, *T*_max_ = 0.984
9323 measured reflections
3349 independent reflections
2520 reflections with *I* > 2σ(*I*)
*R*_int_ = 0.023

#### Refinement

*R*[*F* ^2^ > 2σ(*F* ^2^)] = 0.041
*wR*(*F* ^2^) = 0.119
*S* = 1.04
3349 reflections
223 parameters
1 restraint
H atoms treated by a mixture of independent and constrained refinement
Δρ_max_ = 0.20 e Å^−3^
Δρ_min_ = −0.16 e Å^−3^

### 

Data collection: *SMART* (Bruker, 2002 ▶); cell refinement: *SAINT* (Bruker, 2002 ▶); data reduction: *SAINT*; program(s) used to solve structure: *SHELXS97* (Sheldrick, 2008 ▶); program(s) used to refine structure: *SHELXL97* (Sheldrick, 2008 ▶); molecular graphics: *SHELXTL* (Sheldrick, 2008 ▶); software used to prepare material for publication: *SHELXTL*.

## Supplementary Material

Crystal structure: contains datablocks global, I. DOI: 10.1107/S1600536809043086/jj2010sup1.cif

Structure factors: contains datablocks I. DOI: 10.1107/S1600536809043086/jj2010Isup2.hkl

Additional supplementary materials:  crystallographic information; 3D view; checkCIF report

## Figures and Tables

**Table 1:** Hydrogen-bond geometry (Å, °)

*D*—H⋯*A*	*D*—H	H⋯*A*	*D*⋯*A*	*D*—H⋯*A*
N2—H2⋯O3	0.892 (9)	1.929 (14)	2.6613 (14)	138.3 (16)
O3—H3⋯O2^i^	0.82	1.86	2.6689 (13)	167

Symmetry code: (i) 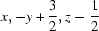.

**Fig. 1.**
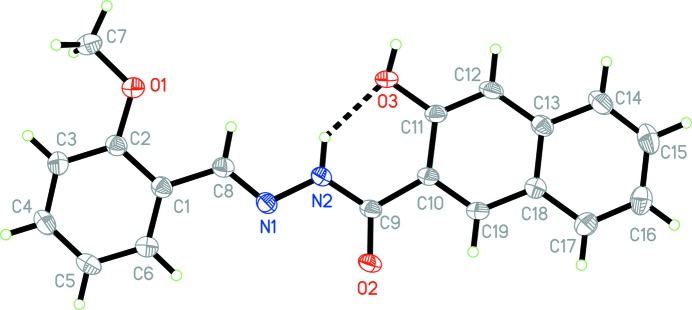
The molecular structure of the title compound with 30% probability ellipsoids. A strong intramolecular N—H···O hydrogen bond is shown as a dashed line.

**Fig. 2.**
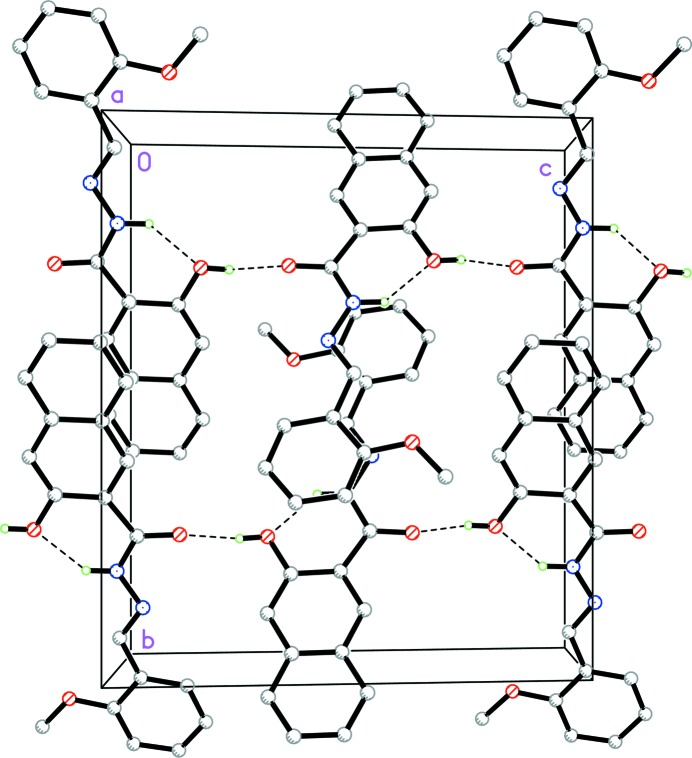
Molecular packing of the title compound. Strong intramolecular N—H···O and intermolecular O—H···O hydrogen bonds (shown as dashed lines) form chains of molecules along the c axis of the unit cell help to provide stability in crystal packing. .

## supplementary crystallographic information

### Comment

Schiff base compounds, important to the pharmaceutical and medicinal fields (Dao *et al.*, 2000; Sriram *et al.*, 2006; Karthikeyan *et al.*, 2006), have been used as versatile ligands in a variety of coordination chemistry applications (Ali *et al.*, 2008; Kargar *et al.*, 2009; Yeap *et al.*, 2009). A number of contributions to these areas have been recently reported (Fun *et al.*, 2009; Nadeem *et al.*, 2009; Eltayeb *et al.*, 2008). With our continued interest in the structural chararcterization of these compounds (Hao, 2009a,b) the title compound, C_19_H_16_N_2_O_3_, (I), is reported.

In the title compound,(I), the mean plane of the hydrazide group, O2/C9/N2/N1/C8, forms dihedral angles of 2.0 (2) and 2.2 (2)°, with the mean planes of the benzene (C1–C6) and naphthyl rings (C10–C19), respectively (Fig. 1). The dihedral angle between the mean planes of the benzene and naphthyl rings is 0.9 (2)°, indicating the planarity of the molecule. All the bond lengths and angles are within normal values (Allen *et al.*, 1987). Crystal packing is enhanced by strong intramolecular N—H···O and intermolecular O—H···O hydrogen bonds (Table 1), forming infinite one-dimensional chains running along the *c* axis of the unit cell (Fig. 2).

### Experimental

2-Methoxybenzaldehyde (0.1 mmol, 13.6 mg) and 3-hydroxy-2-naphthohydrazide (0.1 mmol) were refluxed in a 30 ml methanol solution for 30 min to give a clear colorless solution. Colorless block-shaped single crystals of the compound were formed by slow evaporation of the solvent over several days at room temperature.

### Refinement

H2 was located from a difference Fourier map and refined isotropically, with the N—H distance restrained to 0.90 (1)Å, and with *U*_iso_ restrained to 0.08Å^2^. Other H atoms were constrained to ideal geometries, with d(C—H) = 0.93-0.96 Å, d(O—H) = 0.82 Å, and with *U*_iso_(H) = 1.2*U*_eq_(C) and 1.5*U*_eq_(O3 and C7).

### Crystal data

**Table:**

C_19_H_16_N_2_O_3_	*F*(000) = 672
*M**_r_* = 320.34	*D*_x_ = 1.383 Mg m^−^^3^
Monoclinic, *P*2_1_/*c*	Mo *K*α radiation, λ = 0.71073 Å
Hall symbol: -P 2ybc	Cell parameters from 3028 reflections
*a* = 7.4990 (6) Å	θ = 2.6–30.0°
*b* = 15.4256 (13) Å	µ = 0.10 mm^−^^1^
*c* = 13.3903 (12) Å	*T* = 298 K
β = 96.709 (4)°	Block, yellow
*V* = 1538.3 (2) Å^3^	0.18 × 0.17 × 0.17 mm
*Z* = 4	

### Data collection

**Table:**

Bruker SMART CCD area-detector diffractometer	3349 independent reflections
Radiation source: fine-focus sealed tube	2520 reflections with *I* > 2σ(*I*)
graphite	*R*_int_ = 0.023
ω scans	θ_max_ = 27.0°, θ_min_ = 2.0°
Absorption correction: multi-scan (*SADABS*; Sheldrick, 1996)	*h* = −9→9
*T*_min_ = 0.983, *T*_max_ = 0.984	*k* = −19→19
9323 measured reflections	*l* = −17→14

### Refinement

**Table:**

Refinement on *F*^2^	Secondary atom site location: difference Fourier map
Least-squares matrix: full	Hydrogen site location: inferred from neighbouring sites
*R*[*F*^2^ > 2σ(*F*^2^)] = 0.041	H atoms treated by a mixture of independent and constrained refinement
*wR*(*F*^2^) = 0.119	*w* = 1/[σ^2^(*F*_o_^2^) + (0.0572*P*)^2^ + 0.2454*P*] where *P* = (*F*_o_^2^ + 2*F*_c_^2^)/3
*S* = 1.04	(Δ/σ)_max_ = 0.001
3349 reflections	Δρ_max_ = 0.20 e Å^−^^3^
223 parameters	Δρ_min_ = −0.16 e Å^−^^3^
1 restraint	Extinction correction: *SHELXL97* (Sheldrick, 2008), Fc^*^=kFc[1+0.001xFc^2^λ^3^/sin(2θ)]^-1/4^
Primary atom site location: structure-invariant direct methods	Extinction coefficient: 0.0080 (13)

### Special details

**Table:**

Geometry. All esds (except the esd in the dihedral angle between two l.s. planes) are estimated using the full covariance matrix. The cell esds are taken into account individually in the estimation of esds in distances, angles and torsion angles; correlations between esds in cell parameters are only used when they are defined by crystal symmetry. An approximate (isotropic) treatment of cell esds is used for estimating esds involving l.s. planes.
Refinement. Refinement of *F*^2^ against ALL reflections. The weighted *R*-factor *wR* and goodness of fit *S* are based on *F*^2^, conventional *R*-factors *R* are based on *F*, with *F* set to zero for negative *F*^2^. The threshold expression of *F*^2^ > 2σ(*F*^2^) is used only for calculating *R*-factors(gt) *etc*. and is not relevant to the choice of reflections for refinement. *R*-factors based on *F*^2^ are statistically about twice as large as those based on *F*, and *R*- factors based on ALL data will be even larger.

### Fractional atomic coordinates and isotropic or equivalent isotropic displacement parameters (Å^2^)

**Table:**

	*x*	*y*	*z*	*U*_iso_*/*U*_eq_	
O1	0.12815 (15)	1.07729 (7)	0.87156 (7)	0.0541 (3)	
O2	0.53258 (17)	0.75013 (6)	1.13388 (7)	0.0558 (3)	
O3	0.43652 (16)	0.74188 (7)	0.81939 (7)	0.0526 (3)	
H3	0.4525	0.7391	0.7599	0.079*	
N1	0.36704 (15)	0.89208 (7)	1.04707 (8)	0.0409 (3)	
N2	0.41657 (16)	0.82106 (7)	0.99432 (8)	0.0408 (3)	
C1	0.23017 (18)	1.03244 (9)	1.03531 (10)	0.0394 (3)	
C2	0.14420 (18)	1.09540 (9)	0.97151 (10)	0.0399 (3)	
C3	0.0791 (2)	1.17049 (10)	1.01086 (12)	0.0502 (4)	
H3A	0.0221	1.2122	0.9682	0.060*	
C4	0.0989 (3)	1.18316 (11)	1.11277 (13)	0.0663 (5)	
H4	0.0542	1.2334	1.1391	0.080*	
C5	0.1840 (3)	1.12260 (12)	1.17626 (12)	0.0731 (6)	
H5	0.1976	1.1321	1.2453	0.088*	
C6	0.2491 (2)	1.04788 (10)	1.13819 (11)	0.0553 (4)	
H6	0.3067	1.0071	1.1818	0.066*	
C7	0.0339 (3)	1.13737 (12)	0.80462 (11)	0.0631 (5)	
H7A	−0.0867	1.1436	0.8210	0.095*	
H7B	0.0313	1.1166	0.7369	0.095*	
H7C	0.0934	1.1925	0.8107	0.095*	
C8	0.29350 (19)	0.95271 (9)	0.99292 (10)	0.0422 (3)	
H8	0.2795	0.9460	0.9234	0.051*	
C9	0.49987 (18)	0.75329 (8)	1.04213 (9)	0.0371 (3)	
C10	0.55517 (17)	0.68060 (8)	0.97827 (9)	0.0348 (3)	
C11	0.52607 (18)	0.67543 (9)	0.87108 (9)	0.0381 (3)	
C12	0.5848 (2)	0.60516 (9)	0.82249 (10)	0.0439 (3)	
H12	0.5655	0.6030	0.7526	0.053*	
C13	0.67397 (19)	0.53573 (9)	0.87531 (10)	0.0410 (3)	
C14	0.7369 (2)	0.46152 (10)	0.82747 (12)	0.0561 (4)	
H14	0.7206	0.4577	0.7577	0.067*	
C15	0.8204 (2)	0.39614 (10)	0.88198 (14)	0.0615 (5)	
H15	0.8599	0.3480	0.8490	0.074*	
C16	0.8481 (2)	0.39985 (10)	0.98705 (13)	0.0558 (4)	
H16	0.9060	0.3546	1.0235	0.067*	
C17	0.79016 (19)	0.46984 (9)	1.03570 (11)	0.0459 (3)	
H17	0.8084	0.4720	1.1056	0.055*	
C18	0.70267 (17)	0.53921 (8)	0.98165 (10)	0.0370 (3)	
C19	0.64040 (17)	0.61253 (9)	1.02959 (9)	0.0376 (3)	
H19	0.6579	0.6150	1.0994	0.045*	
H2	0.398 (2)	0.8200 (12)	0.9273 (7)	0.080*	

### Atomic displacement parameters (Å^2^)

**Table:**

	*U*^11^	*U*^22^	*U*^33^	*U*^12^	*U*^13^	*U*^23^
O1	0.0763 (8)	0.0503 (6)	0.0362 (5)	0.0137 (5)	0.0089 (5)	0.0025 (4)
O2	0.0943 (9)	0.0461 (6)	0.0267 (5)	0.0082 (5)	0.0067 (5)	−0.0025 (4)
O3	0.0792 (8)	0.0541 (6)	0.0249 (5)	0.0150 (5)	0.0074 (5)	0.0044 (4)
N1	0.0482 (7)	0.0378 (6)	0.0375 (6)	−0.0002 (5)	0.0085 (5)	−0.0037 (5)
N2	0.0546 (7)	0.0381 (6)	0.0302 (6)	0.0043 (5)	0.0072 (5)	−0.0025 (5)
C1	0.0418 (7)	0.0381 (7)	0.0388 (7)	−0.0053 (6)	0.0063 (6)	−0.0028 (6)
C2	0.0435 (8)	0.0392 (7)	0.0375 (7)	−0.0052 (6)	0.0059 (6)	−0.0016 (6)
C3	0.0600 (9)	0.0390 (8)	0.0502 (8)	0.0048 (7)	0.0007 (7)	−0.0027 (6)
C4	0.0891 (13)	0.0515 (10)	0.0554 (10)	0.0164 (9)	−0.0042 (9)	−0.0200 (8)
C5	0.1083 (15)	0.0666 (12)	0.0402 (9)	0.0207 (11)	−0.0087 (9)	−0.0189 (8)
C6	0.0726 (11)	0.0507 (9)	0.0398 (8)	0.0091 (8)	−0.0047 (7)	−0.0034 (7)
C7	0.0817 (12)	0.0653 (11)	0.0412 (8)	0.0150 (9)	0.0030 (8)	0.0094 (8)
C8	0.0514 (8)	0.0423 (8)	0.0338 (7)	0.0004 (6)	0.0087 (6)	0.0007 (6)
C9	0.0471 (8)	0.0370 (7)	0.0279 (6)	−0.0065 (6)	0.0071 (5)	−0.0007 (5)
C10	0.0398 (7)	0.0376 (7)	0.0274 (6)	−0.0055 (5)	0.0060 (5)	−0.0005 (5)
C11	0.0457 (8)	0.0412 (7)	0.0279 (6)	−0.0021 (6)	0.0063 (5)	0.0036 (5)
C12	0.0595 (9)	0.0474 (8)	0.0261 (6)	−0.0037 (7)	0.0102 (6)	−0.0022 (6)
C13	0.0468 (8)	0.0398 (7)	0.0385 (7)	−0.0054 (6)	0.0137 (6)	−0.0024 (6)
C14	0.0767 (11)	0.0485 (9)	0.0469 (9)	−0.0011 (8)	0.0237 (8)	−0.0075 (7)
C15	0.0700 (11)	0.0409 (9)	0.0788 (12)	0.0036 (8)	0.0303 (9)	−0.0059 (8)
C16	0.0529 (9)	0.0421 (9)	0.0737 (11)	0.0035 (7)	0.0128 (8)	0.0066 (8)
C17	0.0446 (8)	0.0430 (8)	0.0498 (8)	−0.0024 (6)	0.0039 (6)	0.0051 (6)
C18	0.0362 (7)	0.0372 (7)	0.0381 (7)	−0.0056 (5)	0.0068 (5)	0.0009 (5)
C19	0.0427 (7)	0.0415 (7)	0.0280 (6)	−0.0058 (6)	0.0023 (5)	0.0003 (5)

### Geometric parameters (Å, °)

**Table:**

O1—C2	1.3587 (16)	C7—H7B	0.9600
O1—C7	1.4190 (18)	C7—H7C	0.9600
O2—C9	1.2253 (15)	C8—H8	0.9300
O3—C11	1.3683 (16)	C9—C10	1.4978 (18)
O3—H3	0.8200	C10—C19	1.3713 (18)
N1—C8	1.2693 (17)	C10—C11	1.4287 (17)
N1—N2	1.3778 (15)	C11—C12	1.3635 (19)
N2—C9	1.3415 (17)	C12—C13	1.409 (2)
N2—H2	0.892 (9)	C12—H12	0.9300
C1—C6	1.3889 (19)	C13—C18	1.4160 (18)
C1—C2	1.4000 (19)	C13—C14	1.419 (2)
C1—C8	1.4571 (19)	C14—C15	1.355 (2)
C2—C3	1.385 (2)	C14—H14	0.9300
C3—C4	1.369 (2)	C15—C16	1.399 (2)
C3—H3A	0.9300	C15—H15	0.9300
C4—C5	1.369 (2)	C16—C17	1.358 (2)
C4—H4	0.9300	C16—H16	0.9300
C5—C6	1.373 (2)	C17—C18	1.4101 (19)
C5—H5	0.9300	C17—H17	0.9300
C6—H6	0.9300	C18—C19	1.4069 (19)
C7—H7A	0.9600	C19—H19	0.9300
			
C2—O1—C7	117.92 (11)	O2—C9—N2	122.48 (12)
C11—O3—H3	109.5	O2—C9—C10	120.42 (12)
C8—N1—N2	114.72 (11)	N2—C9—C10	117.10 (11)
C9—N2—N1	120.89 (11)	C19—C10—C11	117.92 (12)
C9—N2—H2	118.3 (12)	C19—C10—C9	115.53 (11)
N1—N2—H2	120.7 (12)	C11—C10—C9	126.54 (12)
C6—C1—C2	118.21 (13)	C12—C11—O3	121.40 (11)
C6—C1—C8	122.06 (13)	C12—C11—C10	120.25 (12)
C2—C1—C8	119.71 (12)	O3—C11—C10	118.34 (11)
O1—C2—C3	123.52 (13)	C11—C12—C13	121.71 (12)
O1—C2—C1	116.10 (12)	C11—C12—H12	119.1
C3—C2—C1	120.38 (13)	C13—C12—H12	119.1
C4—C3—C2	119.77 (14)	C12—C13—C18	118.92 (12)
C4—C3—H3A	120.1	C12—C13—C14	123.37 (13)
C2—C3—H3A	120.1	C18—C13—C14	117.71 (13)
C3—C4—C5	120.65 (15)	C15—C14—C13	120.96 (15)
C3—C4—H4	119.7	C15—C14—H14	119.5
C5—C4—H4	119.7	C13—C14—H14	119.5
C4—C5—C6	120.15 (15)	C14—C15—C16	121.09 (15)
C4—C5—H5	119.9	C14—C15—H15	119.5
C6—C5—H5	119.9	C16—C15—H15	119.5
C5—C6—C1	120.84 (15)	C17—C16—C15	119.77 (15)
C5—C6—H6	119.6	C17—C16—H16	120.1
C1—C6—H6	119.6	C15—C16—H16	120.1
O1—C7—H7A	109.5	C16—C17—C18	120.85 (14)
O1—C7—H7B	109.5	C16—C17—H17	119.6
H7A—C7—H7B	109.5	C18—C17—H17	119.6
O1—C7—H7C	109.5	C19—C18—C17	122.36 (12)
H7A—C7—H7C	109.5	C19—C18—C13	118.02 (12)
H7B—C7—H7C	109.5	C17—C18—C13	119.62 (12)
N1—C8—C1	122.61 (12)	C10—C19—C18	123.16 (12)
N1—C8—H8	118.7	C10—C19—H19	118.4
C1—C8—H8	118.7	C18—C19—H19	118.4

### Hydrogen-bond geometry (Å, °)

**Table:**

*D*—H···*A*	*D*—H	H···*A*	*D*···*A*	*D*—H···*A*
N2—H2···O3	0.89 (1)	1.93 (1)	2.6613 (14)	138 (2)
O3—H3···O2^i^	0.82	1.86	2.6689 (13)	167

Symmetry codes: (i) *x*, −*y*+3/2, *z*−1/2.
